# Pharmacological Effects of a Novel Bradykinin-Related Peptide (RR-18) from the Skin Secretion of the Hejiang Frog (*Ordorrana hejiangensis*) on Smooth Muscle

**DOI:** 10.3390/biomedicines8070225

**Published:** 2020-07-17

**Authors:** Xiaowei Zhou, Jie Xu, Ruimin Zhong, Chengbang Ma, Mei Zhou, Zhijian Cao, Xinping Xi, Chris Shaw, Tianbao Chen, Lei Wang, Hang Fai Kwok

**Affiliations:** 1 Institute of Translational Medicine, Faculty of Health Sciences, University of Macau, Avenida da Universidade, Taipa, Macau; xiaoweizhou@um.edu.mo; 2 Natural Drug Discovery Group, School of Pharmacy, Queen’s University, Belfast BT9 7BL, UK; jxu06@qub.ac.uk (J.X.); c.ma@qub.ac.uk (C.M.); m.zhou@qub.ac.uk (M.Z.); chris.shaw@qub.ac.uk (C.S.); t.chen@qub.ac.uk (T.C.); l.wang@qub.ac.uk (L.W.); 3 Department of Nutrition, Henry Fok School of Food Science and Engineering, Shaoguan University, Shaoguan 512005, China; sgu_zrm@sgu.edu.cn; 4 State Key Laboratory of Virology, Modern Virology Research Center, College of Life Sciences, Wuhan University, Wuhan 430072, China; zjcao@whu.edu.cn

**Keywords:** frog, bradykinin related peptide, skin secretion, antagonist, smooth muscle

## Abstract

Bradykinin (BK) and bradykinin-related peptides (BRPs), which were identified from a diversity of amphibian skin secretions, exerted contractile and relaxing effects on non-vascular and vascular smooth muscle, respectively. Here, we report a novel bradykinin-related peptide with a molecular mass of 1890.2 Da, RVAGPDKPARISGLSPLR, which was isolated and identified from *Ordorrana hejiangensis* skin secretions, followed by a C-terminal extension sequence VAPQIV. The biosynthetic precursor-encoding cDNA was cloned by the “shotgun” cloning method, and the novel RR-18 was identified and structurally confirmed by high-performance liquid chromatography (HPLC) and tandem mass spectrometry (MS/MS). Subsequently, the myotropic activity of the synthetic replicate of RR-18 was investigated on the rat bladder, uterus, tail artery and ileum smooth muscle. The peptide was named RR-18 in accordance (R = N-terminal arginine, R = C-terminal arginine, 18 = number of residues). In this study, the synthetic replicates of RR-18 showed no agonist/antagonism of BK-induced rat bladder and uterus smooth muscle contraction. However, it displayed an antagonism of bradykinin-induced rat ileum contraction and arterial smooth muscle relaxation. The EC_50_ values of BK for ileum and artery, were 214.7 nM and 18.3 nM, respectively. When the tissue was pretreated with the novel peptide, RR-18, at the maximally effective concentration of bradykinin (1 × 10^−6^ M), bradykinin-induced contraction of the ileum and relaxation of the arterial smooth muscle was reduced by 50–60% and 30–40%, respectively. In conclusion, RR-18 represents novel bradykinin antagonising peptide from amphibian skin secretions. It may provide new insight into possible treatment options for chronic pain and chronic inflammation.

## 1. Introduction

Bradykinin nonapeptide, RPPGFSPFR, was first reported from *Rana temperaria* frog skin in the 1960s [1]. Subsequently, the bioactive components in the skin secretion of amphibians, especially biologically active peptides, such as antimicrobial peptides and pharmacological peptides, have been extensively studied during the past several decades [2,3]. Bradykinin (BK) and bradykinin-related peptides (BRPs), representing one of the major pharmacological peptides, have been widely isolated and identified in skin secretions of 5 families of amphibians, *Leiopelmatidae*, *Ascaphidae*, *Bombinatoridae*, *Hylidae*, and Ranidae [4,5,6]. BK is regulated by the kallikrein-kinin system (KKS) in mammals [7]; however, the frog skin BK/BRPs are not the products of enzyme catalysis by the KKS. The products were secreted from amphibian skin glands as immune defence peptides. Interestingly, extensive reports on the derivation of BRPs revealed that several amphibian skin BRPs were found in their putative predators, such as birds and snakes, suggesting that BRPs are molecular evolutionary adaptations to species-specific predators.

Recently, more than 100 amphibian BRPs have been reported [8]. However, few Auran species have been reported to secrete BRPs. Additionally, unlike antimicrobial peptides which have been studied extensively, just very few BRPs have been identified in amphibian skin. Apart from that, there is also very little information about BRPs in Ranidae frogs belonging to the *Ordorrana hejiangensis* (*O. hejiangensis*). Furthermore, extensive studies demonstrated that most of the BRPs are BK receptors agonist with a dose-dependent contractile activity on non-vascular smooth muscle. Still, some are antagonists in inhibiting contractility of BK on vascular smooth muscle. For instance, the RVA-Thr6-BK showed vasorelaxant activity on rat arterial smooth muscle but exerted contractile activity on bladder, uterus, and ileum smooth muscle [9]. 

Usually, BK exerts its function in combination with two G protein-coupled receptors families, namely B1 and B2 receptors [10]. B1 receptors are upregulated during tissue damage by pro-inflammatory cytokines and the oxidative stress through the nuclear factor kappa B (NF-κB) pathway [11]. However, B2 receptors were widely distributed in multiple tissues. Recently, B1 and B2 receptors were thought to be involved in many diseases, such as cancer, chronic pain, and diabetes [12,13,14]. For instance, a previous report demonstrated that B1 and B2 receptors were significantly expressed in colorectal cancer cells [15]. Therefore, the development of B1 and B2 receptor antagonists is of great significance in pharmacology or clinical applications. 

Here, we report a novel BRP, RR-18, which was first identified in the skin secretion of Hejiang Frog, *O*. *hejiangensis*, followed by a C-terminal extension sequence VAPQIV. Pharmacological assays revealed that RR-18 displayed an antagonism of bradykinin-induced contraction of the rat ileum and relaxation of arterial smooth muscle. Furthermore, our results indicated that RR-18 exerted its BK inhibition activity by mainly targeting B2 receptors. It has been revealed that activation of BK receptors can induce several downstream signalling pathways involved in inflammatory responses [16] suggesting the unique property of RR-18 may be used in the potential treatment of chronic pain and chronic inflammation.

## 2. Experimental Section

### 2.1. Skin Secretion Acquisition

The specimens of the *O. hejiangensis* were captured, settled and skin secretion was acquired from the dorsal skin as described previously [17]. All the procedures were carried out according to the guidelines in the UK Animal (Scientific Procedures) Act 1986, project license PPL 2694, issued by the Department of Health, Social Services and Public Safety, Northern Ireland. Procedures had been vetted by the Institutional Animal Care and Use Committees (IACUC) of Queen’s University Belfast, and approved on 1 March 2011.

### 2.2. “Shotgun” Cloning of cDNA Encoding RR-18 Biosynthetic Precursor from Skin Secretion

The cDNA encoding RR-18 biosynthetic precursor was evaluated by the “shotgun” cloning method as previously described [17]. A Nested Universal Primer A (NUP A) (Clontech, Palo Alto, CA, USA) and a sense degenerated primer (5′-GAWYYAYYHRAGCCYAAADATGTTCA-3′; W = A + T, Y = C + T, H = A + C + T, R = A + G, D = A + G + T) were subjected to the 3′-RACE reaction, from which the products were cloned and subsequently sequenced. 

### 2.3. Isolation and Structural Characterisation of RR-18 from Skin Secretion

The isolation and structural characterisation of RR-18 from the lyophilised *O. hejiangensis* skin secretion was carried out as previously described [17]. In brief, five mg of lyophilised skin secretion was dissolved in trifluoroacetic acid (TFA) (Sigma-Aldrich, Dorset, UK)/H_2_O (0.05:99.95, *v/v*) followed by centrifugation. The supernatant was eluted by a Cecil CE4200 Adept (Amersham Biosciences, Buckinghamshire, UK) reverse-phase High Performance Liquid Chromatography (RP-HPLC) system. A linear gradient elution was performed using a gradient formed from TFA/H_2_O (0.05:99.95, *v*/*v*) to acetonitrile (ACN) (Sigma-Aldrich, Dorset, UK)/H_2_O/TFA (80.00:19.95:0.05, *v*/*v*/*v*) in 240 min. The fractions were monitored at 214 nm at a flow rate of 1 mL/min. The molecular masses of peptides in the fractions were analysed by matrix-assisted laser desorption/ionisation, time-of-flight mass spectrometry (MALDI-TOF MS) (Thermo Fisher Scientific, San Francisco, CA, USA) using alpha-cyano-4-hydroxycinnamic acid (α-CHCA) (Sigma-Aldrich, Dorset, UK) as the matrix and the putative primary structure of RR-18 was analysed using Sequest algorithm against the self-defined Fasta database in proteome Discoverer 1.0 software (Thermo Fisher Scientific, San Jose, CA, USA).

### 2.4. Solid-Phase Peptide Synthesis of Peptides

RR-18 (RVAGPDKPARISGLSPLR) and BK were synthesised using a Tribute peptide synthesiser (Protein Technologies, Tucson, AZ, USA) according to our previous study [17]. The purity of the peptides (>95%) was determined by RP-HPLC (Cecil, Cambridge, UK). The peptides were further confirmed using an LCQ-Fleet electrospray ion-trap mass spectrometer (Thermo Fisher Scientific, San Francisco, CA, USA).

### 2.5. Myotropic Activity Evaluation on Smooth Muscles

The fractions were rotary dried and then performed to screen the myotropic activity on smooth muscle. The synthetic replicated of RR-18 was used to determine the myotropic activity on rat smooth muscle. Female Wistar rats (250–300 g) were euthanised by CO_2_ asphyxiation based on institutional animal experimentation ethics and the UK animal research guidelines. The endothelium of rat tail artery was removed and the proximal rat tail artery ring was dissected about 2 mm in width and connected to a triangular hook. An approximately 0.5 cm-length ring of ileum was cut. After that, the tissues of rat tail artery, bladder, uterus and ileum were mounted into an organ bath (2 mL) containing a Krebs solution (118 mM NaCl, 1.15 mM NaH_2_PO_4_, 2.5 mM CaCl_2_, 25 mM NaHCO_3_, 4.7 mM KCl, 1.1 mM MgCl_2_, and 5.6 mM glucose). All chemicals were purchased from Sigma-Aldrich (Dorset, UK). The bladder, uterus, artery, and ileum tissues were stretched, maintaining the normal physiological tension of 0.75 g, 0.5 g, 0.5 g, and 0.5 g, respectively. Arteries were pre-contracted with phenylephrine (1 × 10^−5^ M) for 10~20 min to achieve constriction plateaux. A range concentration of synthetic peptides (from 10^−11^–10^−5^ M) was prepared in Krebs solution. Tissues were incubated with peptides in a cumulative manner for at least 5 min before reaching the equilibrium for 20 min. RR-18 (10^−6^ M) was applied for 10 min prior to different concentration of BK (10^−11^–10^−5^ M). The myotropic effects of RR-18 on smooth muscles were recorded using a tension sensor with a PowerLab System (AD Instruments Pty Ltd., Oxford, UK). 

### 2.6. Statistical Analysis

Data was analysed using Prism 6 (GraphPad Software, La Jolla, CA, USA). The mean and standard error of responses were analysed by Student’s T-test and the dose-response curves were constructed using a best-fit algorithm. EC_50_ values were calculated from the normalised curves.

## 3. Results

### 3.1. Molecular Cloning of RR-18 Precursor-Encoding cDNAs

**Figure 1:**
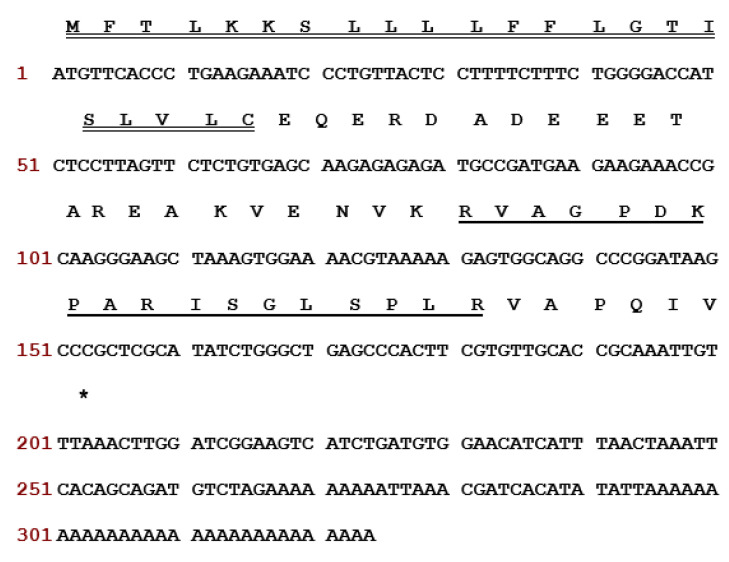
Nucleotide and translated open-reading frame amino acid sequences of the cDNA encoding the biosynthetic precursor of the novel bioactive peptide (RR-18) cloned from hejiang *odorous* frog, *O. hejiangensis*, skin secretion. The putative signal peptide is double-underlined, the mature peptide (RR-18) is single-underlined, and the stop codon is indicated by an asterisk.

The nucleotide and translated open reading frame amino acid sequences of the novel RR-18 precursor encoding cDNAs was cloned from Hejiang Odorous Frog, *O. hejiangensis*, skin secretions are shown in Figure 1. Specifically, the precursor encoding cDNA of the RR-18 contained 67 amino acids including a putative signal peptide domain, an acidic amino acid residue-rich (spacer) domain (21 amino acids), an 18 amino acids length of putative mature peptide and a C-terminal extension peptide domain (-VAPQIV-) (Figure 1). The encoding RR-18 precursor has been deposited in the GenBank Database (accession code: MT522014).

### 3.2. Isolation and Identification and Structural Characterisation of RR-18

**Figure 2:**
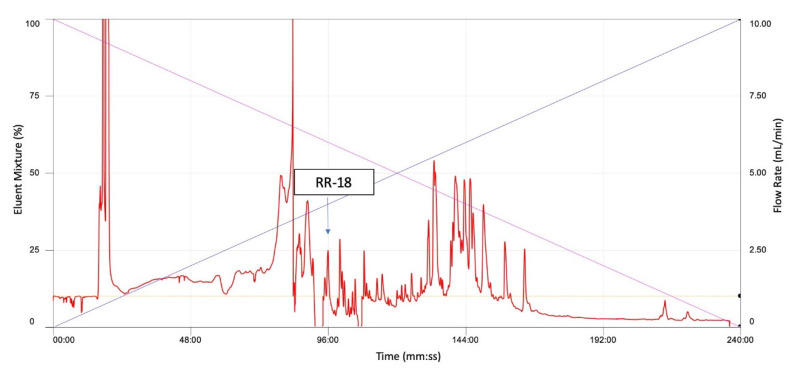
Region of reverse phase HPLC chromatogram of *Odorrana hejiangensis* skin secretion indicating elution position/retention time (arrow) of the novel bioactive peptide. Blue line represented the linear gradient curve of water/acetonitrile/trifluoroacetic acid (TFA) (19.95/80.00/0.05, *v*/*v*/*v*). Pink line represented the linear gradient curve of water/TFA (99.95/0.05, *v*/*v*).

**Figure 3:**
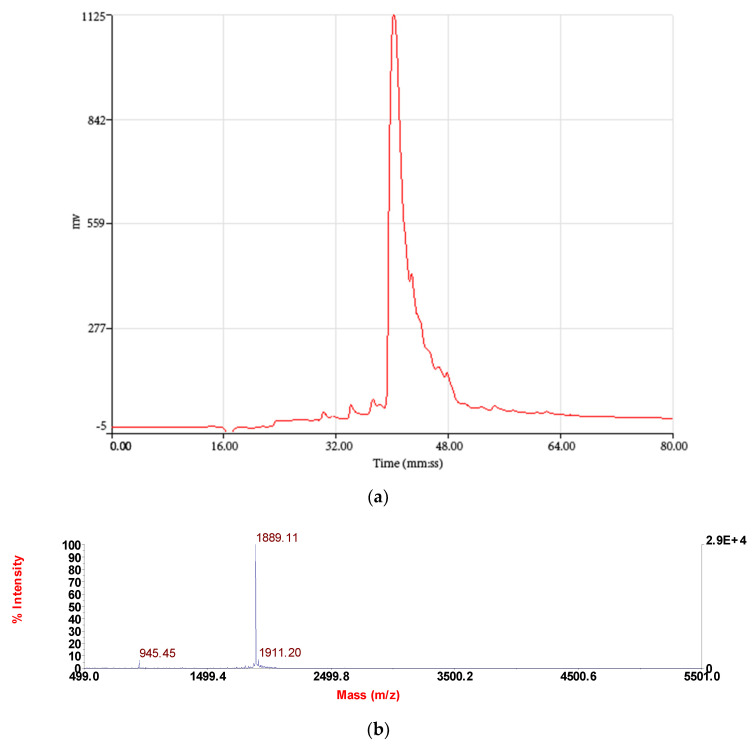
Reverse-phase (RP)-HPLC chromatogram (**a**) and matrix-assisted laser desorption/ionisation, time-of-flight mass spectrometry (MALDI-TOF) mass spectrum (**b**) of a synthetic replicate of peptide RR-18.

**Figure 4:**
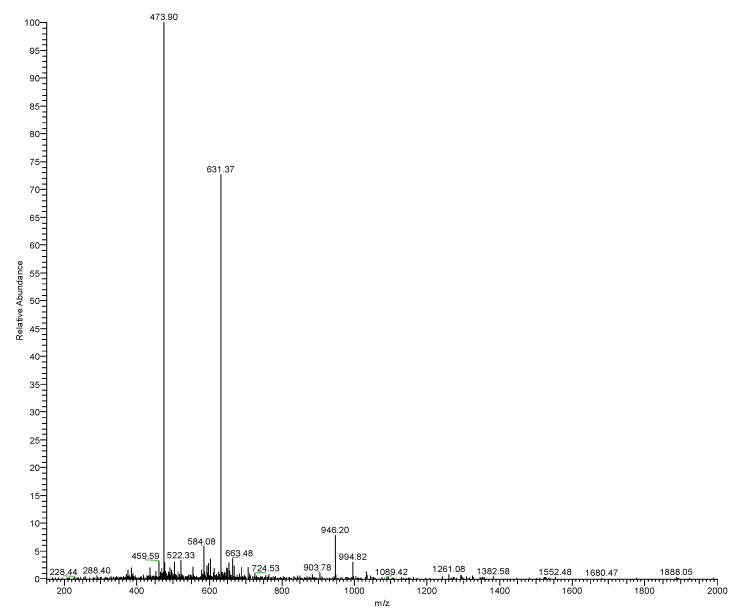
Electrospray MS spectrum of the novel peptide. m/z ion 473.90 is +4 charged (parent 1891.6), m/z ion 631.37 is +3 charged (parent ion 1891.2), and m/z ion 946.20 is +2 charged (parent ion 1890.4). Mean mass of parent 1891.1 vs. calculated of 1890.2 (discrepancy 0.05%).

Reverse-phase HPLC (RP-HPLC) chromatogram of the *O. hejiangensis* skin secretion is shown in Figure 2. Subsequently, the primary structure, RVAGPDKPARISGLSPLR, was thus unequivocally determined by tandem mass spectrometry (MS/MS) fragmentation sequencing (Table 1). The RP-HPLC chromatogram showed that the purity of RR-18 was above 95% and the molecular mass of RR-18 was detected by Matrix-Assisted Laser Desorption/Ionization-Time of Flight (MALDI-TOF) mass spectrum (Figure 3). A single peptide with a mass of 1890.2 Da (Figure 4), which was resolved in HPLC fractions of skin secretion displayed the bradykinin inhibitory activity on smooth muscle.

### 3.3. Bioinformatic Analysis of Novel RR-18

**Figure 5:**
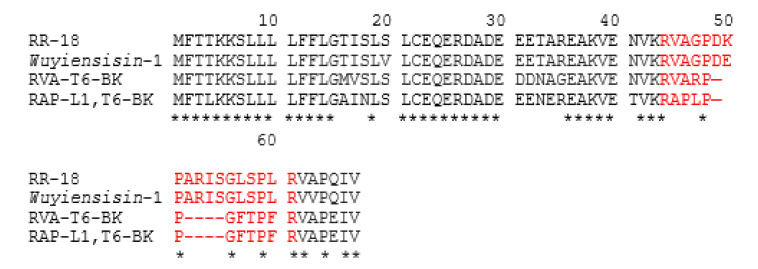
Amino-acid sequence alignment of RR-18 precursor and other bradykinin-related peptide (BRP) precursors from the skin secretion of several frog species. The sequences of mature peptides were labeled in red and stars (*) represented the identical amino acid residues.

BLAST analysis of RR-18 was performed using the National Center for Biotechnological Information (NCBI) on line portal, and demonstrated that the full length open reading frame of RR-18 displayed relative high amino acid sequence identity with the *wuyiensisin*-1 and other typical bradykinin antagonist (RVA-T6-BK and RAP-L1, T6-BK) precursor sequences. Specifically, the similarity between the primary sequence of RR-18 and *wuyiensisin*-1 was over 94%. Additionally, the primary structure of RR-18 showed high similarity with some other typical bradykinin antagonists. The highly-conserved amino acids are Arg^1^, Pro^5^, Pro^8^, Gly^13^, Pro^16^, and Pro^18^ (Figure 5).

### 3.4. Pharmacological Effects of RR-18 on Smooth Muscle

**Figure 6:**
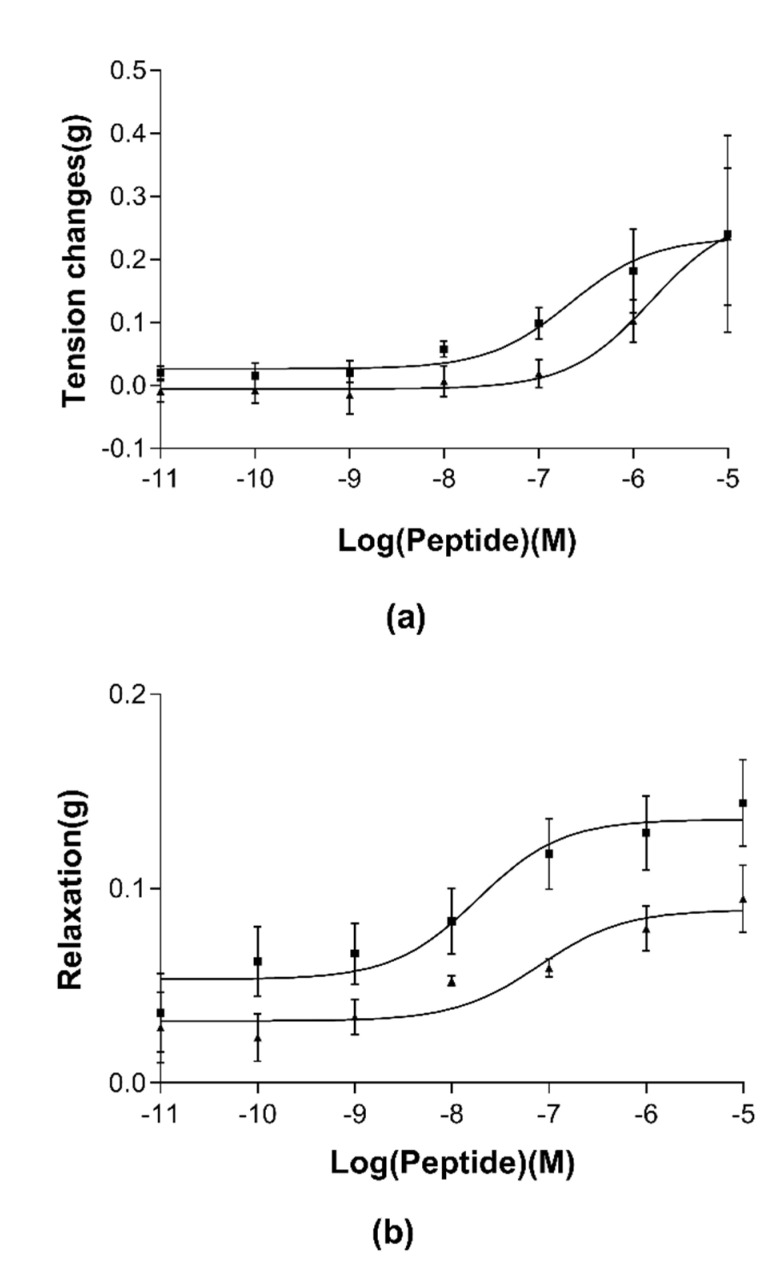
Dose-response curves of contraction effect on rat ileum (**a**) and relaxation effects on a rat tail artery (**b**) smooth muscle preparation in the presence of bradykinin (BK) (■) or the presence of BK with RR-18 (▲).

The purified RR-18 and BK were employed in the evaluation of myotropic activity on rat uterus, bladder, ileum and tail artery. Specifically, RR-18 produced no distinct myotropic action on rat bladder, ileum, uterus and tail artery in its own right. Additionally, RR-18 showed no antagonism of BK-induced rat bladder and uterus smooth muscle contraction. BK produced a dose-response curve in affecting rat ileum and rat tail artery. However, RR-18 mediated a potent inhibition of bradykinin-induced contraction and relaxation of rat ileum and tail artery smooth muscle (Figure 6). Specifically, the EC_50_ values of BK on rat ileum and tail arteries were 214.7 nM and 18.3 nM, respectively. However, when the tissue was pretreated with the novel peptide, RR-18, at the maximally effective concentration of bradykinin (1 × 10^−6^ M), BK-induced contraction of the ileum and relaxation of the arterial smooth muscle was abolished by 50–60% and 30–40%, respectively. Additionally, the EC_50_ values of BK+RR-18 (10^−6^ M) on rat ileum and tail arteries were 1.54 µM and 79.83 nM, respectively. Moreover, RR-18 represented a typical BK competitive inhibitor and non-competitive inhibitor on rat ileum and tail artery smooth muscle, respectively.

## 4. Discussion

Amphibian skin secretions contain a variety of active substances including peptides, proteins, steroids and alkaloids. In particular, peptides, such as bombesin and bradykinin, act as myotropic peptides and are critical to protecting amphibians from predators [8,18]. At the same time, BK and BRPs are associated with many diseases, such as chronic pain and cancer [13,19]. Recently, very few peptides from the Hejiang frog have been reported [9,20,21].

In this study, a novel octade-peptide, RR-18, was identified by de novo sequencing and isolated from the skin secretion of the *O. hejiangensis*. Obviously, *O. hejiangensis* showed high homology compared with the precursors of *Amolops wuyiensis*. Additionally, the precursor encoding cDNA library of RR-18 was further determined. Interestingly, sequence analysis exhibited high similarity (up to 94.4%) between RR-18 and *wuyiensisin*-1 (RVAGPDEPARISGLSPLR-OH; AIU99945.1) which was identified from the Sanchiang sucker frog (*Amolops wuyiensis*). It was noted that the *O. hejiangensis* is a unique amphibian species in China, mainly distributed in the Sichuan, Guangxi, and Chongqing areas of China. At the same time, the *Amolops wuyiensis* are only found in the Fujian, Zhejiang, and Anhui areas of China. This may explain why the same group of peptides could be found in different species in similar regions [22], and this may be of benefit to the evolution of amphibians. Thus, the Hejiang frog and the Sanchiang sucker frog, at least with respect to their skin BRPs, appear to be more closely related to one another than to other ranid species that occupy similar geographical distributions [5]. The residue was substituted to a lysine (K) residue at position 7 from the N-terminal of the peptide.

Additionally, it is significant that RR-18 has a similar structure to that of BK, although its biological activity has not been determined. Besides, compared with another BRP, RVA-Thr6-BK, which was identified in the skin secretion of *O. hejiangensis*, as well [9], the differences between RR-18 and RVA-Thr6-BK are mainly reflected in the N-terminal region. Specifically, an R residue was submitted to G at position 4 from N-terminal. Interestingly, the commutation of a single amino acid residue in wasp kinins can result in significant differences in the action of the peptide. The factors that distinguished the encoded proprepeptides of RR-18 in this family are unclear. This phenomenon may be due to differences in species, regions and the living environments of frogs [5].

Unlike the majority of previously cloned ranid frog skin BRP precursors, which always encode multiple peptides [5], RR-18 only contained a single copy in the proprepeptides. Additionally, the propeptide convertase cleavage site(s) of RR-18 was found to be unusual when compared with other kininogens. Firstly, an acidic amino acid-rich spacer peptide domain of RR-18 has no typical prepropeptide convertase processing site (-KR-). Secondly, the N-processing sites of mature kinin are always RR and KR. However, the N-processing sites of RR-18 are KK and an R. The C-terminus of the mature kinin is flanked by the sequence -VAPQIV- that is cleaved from the maturing kinin by a post arginyl cleavage. These phenomena may be attributed to evolution amongst amphibians [5]. 

Many vasodilators exerted relax activity on rat artery smooth muscle, which is mediated by the endothelium and the release of nitric oxide (NO) [23]. However, previous studies demonstrated that arterial smooth muscle preparations which were pretreated with specific endothelium nitric oxide synthase (eNOS) inhibitor failed to cause significant effects on the dose-responsive relaxation of vasorelaxant indicating that BRPs exert the relaxing activity on rat artery smooth muscle is unlikely to involve the action of NO [24,25]. Our data showed that RR-18 displayed an antagonism of bradykinin-induced rat ileum contraction and arterial smooth muscle relaxation, which is consistent with previous studies, in that Phe at the penultimate position of BRP is the crucial site for activating BK receptors. The Phe at the penultimate position of BRP substituted with Leu could induce an antagonistic activity [26]. Interestingly, RR-18 represented a BK competitive inhibitor and non-competitive inhibitor on rat ileum and tail artery smooth muscle. Firstly, the EC_50_ values of BK and BK+RR-18 in rat tail artery smooth muscle were virtually identical. Secondly, the Emax value of BK+RR-18 is lower to that of BK. However, the Emax values of BK and BK + RR-18 in rat ileum were overlapping, suggesting BK occupied receptor in ileum when the concentration of BK is increasing. Henceforth, RR-18 was overcome by BK in rat ileum. Apart from these, in comparison with original BK, the primary structure of RR-18 displayed multiple segment insertion sites, like -VAG-, -DE-, and -ARIS-, that were inserted between RP, PP, and PG, respectively. Due to RR-18 having exerted different BK antagonism on rat ileum and tail artery, we speculated that the changes of BRPs structure caused various kinds of ligand receptor binding pathways, which affected the pharmacological activity of BRPs [27]. Additionally, a previous study demonstrated that BK exerted its activity by regulating B1 and B2 receptors, especially mainly regulated by B2 receptor [24]. Moreover, RR-18 showed high similarity to some B2 receptor antagonists like RVALPPGFTPLR, QIPGLGPLR and RVA-Thr6-BK [9,24,28], henceforth, we speculated that RR-18 exerted its BK inhibition activity by mainly targeting B2 receptors. Nevertheless, further studies are required to determine whether B1 receptor interaction could explain the competition/non-competition features of RR-18 on ileum and tail artery. Previous reports revealed that Arg1, Pro2, Gly4, Phe5, Pro7, Phe8 and Arg9 are key residues for the biological activity of BK [29]. In this study, both Phe5 and Phe8 were substituted to leucine, which may improve/reduce the affinity between the peptides and receptors or even change an agonist to antagonist [30].

Taken together, a novel BRP, which contains 18 amino acids, was first isolated and identified from *O. hejiangensis*. The cDNA-encoded biosynthetic precursor of RR-18 showed high similarity with a BRP peptide (*wuyiensisin*-1) which was identified from *Amolops*. Our study demonstrated that RR-18 displayed competitive and non-competitive inhibition of BK on rat ileum and rat tail artery smooth muscle, respectively. Furthermore, RR-18 showed BK antagonist activity by mainly activating the B2 receptor. There is compelling evidence that BK is involved in the development of many diseases, such as pain and hyperalgesia [31,32]. Meanwhile, more recently, it has been suggested that BK B2 receptors were upregulated after a traumatic brain injury (TBI) and the inflammatory response was significantly reduced after treatment with a bradykinin B2 receptor inhibitor [33]. Hence, RR-18, as a BK B2 receptor antagonist, may have the potential for developing new drugs for chronic pain and chronic inflammation. Furthermore, the structural diversity of BRPs and its related BK inhibitory activity suggested that anuran is a rich source for the study of the structure-activity relationships between BRPs. Moreover, in addition to canonical research on the fossil record and morphological characteristics, with the in-depth study of molecular techniques and the precursor encoding cDNA sequencing of orthologous genes, it may give us a new understanding of the scope of evolution of amphibians.

## Figures and Tables

**Table 1:** Predicted tandem mass spectrometry (MS/MS) fragmentation *b-* and *y-*ion series ion series (singly-, doubly-, and triply-charged) of RR-18. Observed ions were shown in bold italic typeface.

#1	b(1+)	b(2+)	b(3+)	Seq.	y(1+)	y(2+)	y(3+)	#2
1	157.10840	79.05784	53.04098	R				18
2	256.17682	128.59205	86.06379	V	1734.00218	***867.50473***	***578.67224***	17
3	***327.21394***	164.11061	109.74283	A	1634.93376	***817.97052***	***545.64944***	16
4	***384.23541***	***192.62134***	128.74999	G	1563.89664	***782.45196***	***521.97040***	15
5	***481.28818***	241.14773	161.10091	P	1506.87517	***753.94122***	502.96324	14
6	***596.31513***	***298.66120***	199.44323	D	1409.82240	***705.41484***	470.61232	13
7	***724.41010***	***362.70869***	242.14155	K	***1294.79545***	***647.90136***	432.27000	12
8	***821.46287***	***411.23507***	274.49247	P	1166.70048	***583.85388***	389.57168	11
9	***892.49999***	***446.75363***	***298.17151***	A	***1069.64771***	***535.32749***	357.22075	10
10	1048.60111	***524.80419***	350.20522	R	***998.61059***	***499.80893***	333.54171	9
11	***1161.68518***	***581.34623***	387.89991	I	***842.50947***	***421.75837***	***281.50801***	8
12	1248.71721	***624.86224***	416.91059	S	***729.42540***	365.21634	243.81332	7
13	1305.73868	***653.37298***	***435.91774***	G	***642.39337***	321.70032	***214.80264***	6
14	1418.82275	***709.91501***	473.61243	L	***585.37190***	***293.18959***	195.79548	5
15	1505.85478	***753.43103***	502.62311	S	***472.28783***	***236.64755***	158.10079	4
16	1602.90755	***801.95741***	***534.97403***	P	***385.25580***	***193.13154***	129.09012	3
17	1715.99162	***858.49945***	572.66872	L	288.20303	144.60515	96.73919	2
18				R	175.11896	88.06312	59.04450	1
